# Lessons Learned From the Capacity-Building and Mentorship Program to Improve Health Information Systems in 11 Districts of Ethiopia

**DOI:** 10.9745/GHSP-D-21-00690

**Published:** 2022-09-15

**Authors:** Hiwot Belay, Afrah Mohammedsanni, Abebaw Gebeyehu, Hibret Alemu, Naod Wendrad, Biruk Abate, Wubshet Denboba, Frehiwot Mulugeta, Shemsedin Omer, Barbara Knittel

**Affiliations:** a John Snow, Inc., Ethiopia Data Use Partnership, Addis Ababa, Ethiopia.; b Ethiopia Ministry of Health, Addis Ababa, Ethiopia.; c John Snow, Inc., Arlington, VA, USA.

## Abstract

**Introduction:**

Health information systems (HIS) performance in Ethiopia is currently insufficient, and improvements are required to ensure that decision making is data driven. We share our experiences from the early-stage implementation of a package of HIS capacity-strengthening interventions as part of an innovative academic-government collaboration that addresses challenges in HIS performance.

**Methods:**

We used routine program data to assess HIS performance using the Performance of Routine Information System Management (PRISM) assessment tools. The assessment employed a pre-post design and was conducted in a total of 24 selected health facilities (6 hospitals and 18 health centers) from 11 districts in Ethiopia at project baseline (2018) and midline (2020).

**Results:**

Source document completeness rate reached less than 80% for the majority of the assessed data elements. Improvements were observed in quarterly report completeness (26% vs. 83%) and timeliness (17% vs. 48%). Though data inaccuracies are noted for all assessed data elements in 2020, the majority (83%) of skilled birth attendance and HIV reports (68%) fall within the acceptable range of reporting accuracy. The identification of performance-related problems, using performance monitoring team (PMT) meetings, improved between 2018 and 2020 (67% vs 89%). Similar improvements were also observed in developing action plans to solve identified problems via the PMT (52% in 2018 vs. 89% in 2020). Data use for planning and target setting (65% in 2018 vs. 90% in 2020), reviewing performance (58% in 2018 vs. 60% in 2020), and supervision (51% in 2018 vs. 53% in 2020) all improved among assessed health facilities.

**Discussion:**

This study showed that a capacity-building and mentorship program that engages experts from multiple disciplines and sectors can improve the quality and use of health data. This partnership enabled engagement between government and academic stakeholders and allowed for a more robust exchange of resources and expertise toward HIS improvement.

## Abstract (precis)

Health information systems strengthening interventions, such as the capacity-building and mentorship program used, leverages the expertise of stakeholders from multiple disciplines and can help improve data quality and information use at health facilities.

## Teaser

**Key Findings:**

- As part of a collaborative capacity-building and mentorship program between 6 universities and the Ministry of Health, health facilities observed an improvement in the quality and use of health information system data across several tracer indicators.
- Establishing performance monitoring team meetings encouraged a data use culture for reviewing performance, identifying and solving problems, planning, and setting targets.
- Engaging a diverse group of experts from academia, regional health bureaus, and district health offices to build health worker capacity through training and mentorship was beneficial for improving the quality and use of health data.

**Key Implication:**

- An academic-government collaborative capacity building and mentorship program is a promising approach to improving health information systems performance at the point of health care delivery. Building local ownership of the program, creating local mentors within the existing health system structure, and strengthening HIS expertise at universities are all essential for sustaining and expanding this model to other districts.

## INTRODUCTION

Global priority agendas have identified strengthening health systems as a way to achieve universal health coverage.^1^ Strong health systems rely on the generation and use of relevant, timely, and accurate health data at all levels.^2^ Health sector leaders need quality data to maximize the efficient use of scarce resources to improve performance and track progress towards goals.^2^ Inadequate data quality limits the confidence and value that decision makers place on data and diminishes future demand for data in decision-making processes.^3^^,^^4^ Many health systems fail to fully link evidence to decisions and suffer from an inadequate ability to respond to priority health needs.^2^^,^^5^^,^^6^ The systematic use of data for decision making is critical, as it contributes to the quality and equity of health care delivery and maximizes operational efficiencies.^3^

Over the past 20 years, tremendous efforts have been made to strengthen health information systems (HIS) in low- and middle-income countries (LMICs).^4^ Since 2008, the Ethiopian Ministry of Health (MOH) has undertaken a series of HIS reforms aimed to standardize and improve health data monitoring in the public health sector.

Recognizing the potential of routine health information to guide improvements in quality health care delivery, in 2016, the Government of Ethiopia launched the Information Revolution (IR) Roadmap as a core strategy of the Health Sector Transformation Plan (HSTP).^7^^,^^8^ The IR focuses on cultivating an information culture by digitizing and scaling up priority HIS interventions and strengthening HIS governance. Under the IR, the MOH has worked to standardize and digitize indicators, data collection, and transmission tools, as well as processes that facilitate data generation, handling, and use at the point of care and in management settings.^7^^–^^9^ As a result, the country has made important strides toward establishing an effective, simplified, and harmonized HIS.

To further translate this IR into action, in 2016, the MOH introduced the Connected Woreda Strategy (CWS).^10^ This strategy aims to transform the data use culture and improve data quality through a tiered pathway, directing data use interventions toward primary health facilities and districts (known as “woreda” in Ethiopia).^10^ A seminal activity under the CWS is the Capacity Building and Mentorship Program (CBMP), an academic-government partnership that began implementation in 2017. Under the CBMP, local universities provide regional health bureaus (RHBs) and zonal health departments (ZHDs) with opportunities to build their data quality and use capacities, following up with technical assistance. The universities also support the RHBs in implementing a package of capacity-building interventions at the health facility and district levels to improve HIS performance and advance health facilities and districts toward “model” status (Box).

BOX **The Connected Woreda Strategy**

The Connected Woreda is a strategy that provides a tiered pathway to achieve the highest levels of data quality and use at health facility and district levels. The strategy begins by evaluating and scoring facilities against a common set of criteria related to HIS structure and resources (30%), data quality (30%), and data use (40%). Facilities that score less than 65% are categorized as “emerging,” those between 65% and 90% are categorized as “candidate,” and those above 90% are accredited as a “model facility.” The grading process is followed by the development and implementation of action plans tailored to address identified barriers to data quality and use.

Under the CBMP, local universities provide RHBs and ZHDs with opportunities to build their data quality and use capacities, following up with technical assistance.

To support the MOH in addressing HIS challenges at a national scale, since 2016, the Ethiopia Data Use Partnership (DUP)—a consortium of technical partners committed to improving the collection and use of high-quality routine information in the health sector—has partnered with the MOH to advance the IR’s data use goals. The DUP supports HIS digitization, improves governance, enhances implementation research and knowledge management, and addresses other aspects of engendering a culture of health systems data use. DUP supports this work while stressing country leadership, ownership, and strong coordination among stakeholders from multiple disciplines and sectors. In partnership with 6 local universities, DUP is also supporting the implementation of the CBMP across Ethiopia.

We provide experiences and lessons learned from the initial implementation of the CBMP at the health facility and district levels in Ethiopia to inform program improvements, optimize implementation of the IR agenda, and maximize its impact.

## CBMP INTERVENTION DESCRIPTION

The CBMP is currently being implemented in 187 public primary health centers and hospitals in 36 districts and 3 subcities located across all regions in Ethiopia. Under the CBMP, 6 universities (Addis Ababa University, Haramaya University, Hawassa University, Jimma University, Mekelle University, and the University of Gondar) are supporting RHBs as they roll out a package of capacity-building interventions at district and facility levels. The package includes supportive supervision; on-site coaching and mentorship; training in data quality, analysis, visualization, and use; cross-site experience sharing; quality improvement initiatives, such as using performance monitoring teams (PMTs); and other activities designed to develop the skills and knowledge base of health workers and managers in data quality and information use. Interventions were adapted and tailored to each site based on ongoing monitoring via a routine (quarterly) needs assessment.

The partnership with local universities also serves to progress organizational capacity building by linking academia to program implementation. University collaborators engaged in evidence generation and publication on themes related to the CWS implementation and, to date, have provided 81 Master’s and 8 PhD students the opportunity to conduct their theses on these topics. Moreover, the universities also conduct research within these sites to explore effective ways of implementing HIS interventions and improving HIS performance.

### Learning and Demonstration Sites

A subset of 74 CBMP program sites were selected by DUP partners and RHB collaborators to become “learning and demonstration sites.” These sites received additional resources and more intensive capacity-building support to develop and implement site-specific HIS strengthening plans. They also received more frequent and structured mentorship visits (i.e., every 2 months) and on-site needs-based trainings focused on improving HIS capacity and competency among staff. As an initial step of a long-term strategy to diffuse learnings to other districts and health facilities across the regions, these sites served as incubation and testing locations for data use interventions.

## METHODS

### Study Design

This study assesses the effect of the more intensive CBMP HIS intervention package implemented in the learning and demonstration sites on HIS performance at the primary facility level. We measured HIS performance at 24 learning and demonstration sites in 2018, then compared these indicators against the same measures at the same sites in 2020. The 24 sites (6 hospitals and 18 health centers) were randomly selected from the 74 CBMP learning and demonstration sites. The selected sites represent 1 district within each of the 11 regions in Ethiopia.

The Performance of Routine Information System Management (PRISM) tools^11^ were used to measure the effect of the CBMP intervention package on HIS performance in selected study sites. Specifically, the following PRISM assessment tools were adapted to the local context and used for this study: District Routine Health Information System (RHIS) Performance Diagnostic Tool; Facility Level RHIS Performance Diagnostic Tool; Management Assessment Tool; and Facility/Office Checklist. The PRISM tools provide a structured way to assess RHIS data quality and information use in health facilities and support the identification of strengths and weaknesses in these areas that can inform intervention refinements.^12^

The data collection team received an intensive 5-day training, guided by a structured training manual, led by experts who were familiar with the Ethiopian health system and PRISM tools. Data were collected using tablets loaded with SurveyCTO, an Android-based mobile data collection platform, by data collectors who had experience in similar activities and were familiar with the Ethiopian health system. Internal data quality validation rules were programmed in SurveyCTO to minimize data collection errors. The data were uploaded in real time and managed on a cloud server. Regular data checks were administered throughout data collection to ensure quality.

### Data Analysis

In line with the World Health Organization’s (WHO) data quality assessment guideline, 7 tracer indicators were selected by the MOH to assess HIS data quality over a 3-month period in 2018 and 2020: (1) number of births attended by skilled birth attendant, (2) number of new and repeat contraceptive acceptors, (3) children younger than age 1 year who have received a third dose of the pentavalent vaccine (penta 3), (4) number of clients who tested positive for HIV, (5) number of children younger than age 5 years with pneumonia who received treatment (U5 pneumonia), (6) number of confirmed malaria cases using microscopy or rapid diagnostic testing, and (7) number of all forms of TB cases (Table 1).^11^^,^^13^ The PRISM tools measure HIS data quality in 3 dimensions: (1) data completeness, (2) reporting timeliness, and (3) data accuracy.

**TABLE 1.** Facility-Level Tracer Indicators Selected by the Ministry of Health to Assess HIS Data Quality, Ethiopia

**Indicators**	**Reportable Data Elements at Facility Level**
Proportion of births attended by skilled attendant	Number of births attended by skilled attendant
Contraceptive acceptors rate	Number of new and repeat contraceptive acceptors
Pentavalent 3 immunization coverage	Number of children under age 1 year who have received 3 doses of pentavalent vaccine
Clients who tested positive for HIV	Number of clients who tested positive for HIV
Proportion of children under 5 years of age with pneumonia who received treatment	Number of children under age 5 years with pneumonia who received treatment
Malaria diagnostic testing rate	Number of cases of malaria confirmed by microscopy or RDT
TB case detection rate for all forms of TB	Number of all forms of TB cases detected

Abbreviation: HIS, health information system; RDT, rapid diagnostic test.

1. **Data completeness**
  - **Source document completeness** (facility level): Number of facilities that completed primary source documents (e.g., registers and tally sheets).
  - **Reporting completeness** (district level): Number of expected reports compared to the number of reports actually submitted to the district health office.
2. **Reporting timeliness** (district level): Number of facility reports received by the district health office by the predetermined deadline.

**Data accuracy:** Numerical consistency between the data recorded in facility source documents and the data aggregated in monthly reports submitted to the district health office. Report accuracy was assessed among facilities providing services associated with the 7 tracer indicators (Table 1) reviewed in this study. In this article, we used the MOH and WHO’s “acceptability tolerance range” for discrepancies in data accuracy (i.e., within ±10%).^11^^,^^13^

The PRISM tools were also used to assess HIS performance related to information use by measuring the use of data for planning and target setting; performance review and evidence-based decision-making; production of analytic reports; and data dissemination. The PRISM tools were also applied to assess the status of HIS data management and processing related to data quality assurance practices, data analysis and visualization, supervision, and feedback mechanisms.

Data were cleaned and analyzed using Stata version 14.0 and are presented as descriptive statistics based on MEASURE Evaluation’s PRISM analysis guideline.^14^

### Ethics Approval

Ethical approval was sought from and granted by the Ethiopian Public Health Association EPHA/OG/203/20.

## RESULTS

### HIS Data Quality

#### Source Document Completeness

The overall source document completeness rate (i.e., facilities with complete source documents for all 3 assessed months) increased from 67.9% to 75.3% between 2018 and 2020. The proportion of health facilities with complete source documents increased from 2018 to 2020 across most tracer indicators: penta 3 (73% to 81%), contraceptive acceptors (67% to 87%), U5 pneumonia (58% to 83%), and TB (79% to 83%) (Table 2).

**TABLE 2.** Changes in Facility-Level Completeness of Data Elements in Source Documents Between 2018 and 2020, by Tracer Indicator, Ethiopia

**Indicators**	**2018**		**2020**	
	**Total No. Facilities**	**No. (%)**	**Total No. Facilities**	**No. (%)**
Proportion of births attended by skilled birth attendant	24	16 (66.7)	24	17 (70.8)
Third dose of pentavalent immunization coverage	22	16 (72.7)	21	17 (81.0)
Contraceptive acceptors rate	24	16 (66.7)	23	20 (87.0)
Clients who tested positive for HIV	24	18 (75.0)	24	15 (62.5)
Malaria diagnostic testing rate	23	13 (56.5)	22	13 (59.1)
Proportion of children aged under 5 years with pneumonia who received treatment	24	14 (58.3)	24	20 (83.3)
TB case detection rate for all types of TB	24	19 (79.2)	24	20 (83.3)

By 2020, HIS data quality improved in source document completeness, reporting completeness and timeliness, and reporting accuracy.

#### Reporting Completeness and Timeliness

Reporting completeness and timeliness for each report type that facilities were expected to submit (i.e., service reports, outpatient department visits, and quarterly reports) improved between 2018 and 2020 across all assessed report types (Figure). Improvements in completeness and timeliness were most notable for quarterly reports, which improved from 26% to 83% and 17% to 48%, respectively.

**FIGURE:**
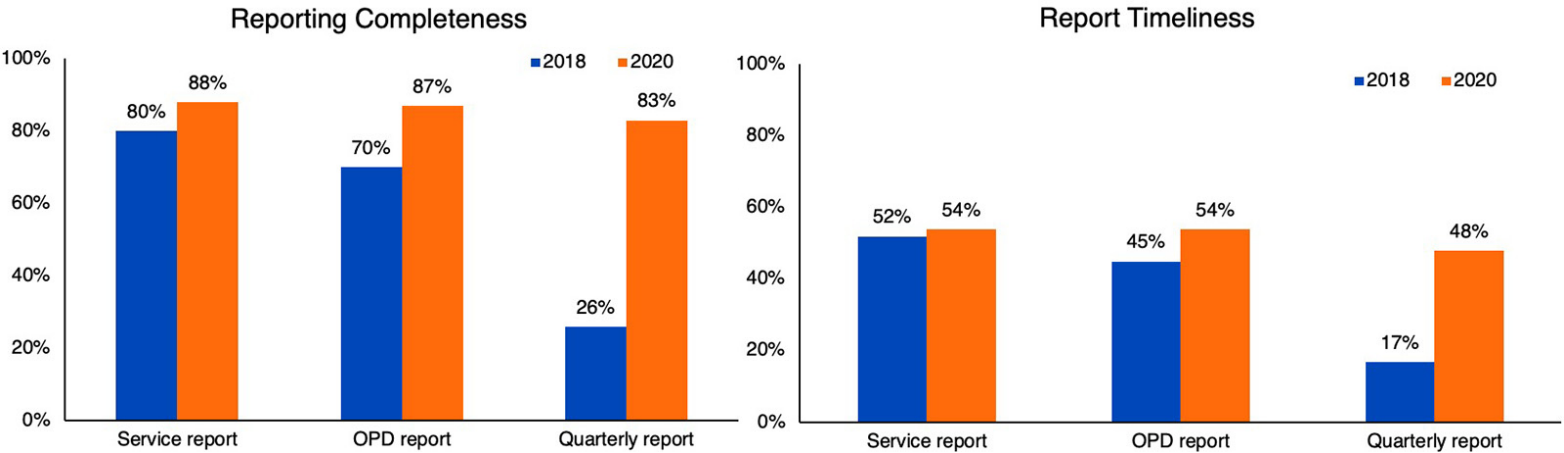
Changes in Reporting Completeness and Timeliness of Health Facilities Between 2018 and 2020, by Report Type, Ethiopia Abbreviation: OPD, outpatient department.

#### Reporting Accuracy

During the 2-year study period, the percentage of health facilities that fell within the acceptable data accuracy tolerance improved (Table 3) for nearly all trace indicators. Positive changes in data accuracy were most notable for contraceptive acceptors (48% to 63%), clients who tested positive for HIV (68% to 83%), and confirmed malaria cases (55% to 73%).

**TABLE 3.** Changes in Reporting Data Accuracy^a^ for Health Facilities From 2018 to 2020, by Tracer Indicator, Ethiopia

**Indicators**	**2018**		**2020**	
	**Total No. Facilities**	**Facilities With Acceptable Data Reporting, No. (%)**	**Total No. Facilities**	**Facilities With Acceptable Data Reporting, No. (%)**
Proportion of births attended by skilled birth attendant	23	19 (82.6)	24	20 (83.3)
Third dose of pentavalent immunization coverage	21	12 (57.1)	21	12 (57.1)
Contraceptive acceptors rate	23	11 (47.8)	24	15 (62.5)
Clients who tested positive for HIV	22	15 (68.2)	24	20 (83.3)
Malaria diagnostic testing rate	22	12 (54.6)	22	16 (72.7)
Proportion of children aged under 5 years with pneumonia who received treatment	22	10 (45.5)	24	10 (41.7)
TB case detection rate for all types of TB	20	14 (70.0)	24	18 (75.0)

a Acceptable margin of error is 90%–110%.

### PMT Functionality

All health facilities assessed in this study had PMTs established in 2018 (Table 4). However, only two-thirds of the facilities reported conducting regular PMT meetings (e.g., 1 or more meetings per month), which stayed relatively consistent between 2018 and 2020. The results show improvements from 2018 to 2020 in routine data use for identifying performance-related issues (67% to 89%), conducting performance root cause analyses (62% to 72%), and developing action plans to solve the identified issues (52% to 89%).

**TABLE 4.** Changes in PMT Functionality in Health Facilities Between 2018 and 2020, Ethiopia

Indicators	**2018**		**2020**	
	**Total No. Facilities**	**No. (%)**	**Total No. Facilities**	**No. (%)**
HFs with PMT	24	24 (100)	24	24 (100)
HFs that held regular PMT meetings (3 or more during the assessed 3 months)	24	16 (66.7)	24	15 (62.5)
HFs that have PMT meeting minutes (available for at least 1 meeting held during the assessed 3 months)	22	21 (95.5)	20	18 (90.0)
PMT meetings chaired by facility in-charge/medical director	21	15 (71.4)	18	13 (72.2)
HFs discussed performance targets or tracking progress against target	21	20 (95.2)	18	18 (100)
HFs identified performance-related problems	21	14 (66.7)	18	16 (88.9)
HFs conducted performance root cause analysis	21	13 (61.9)	18	13 (72.2)
HFs developed action plan for performance improvement	21	11 (52.4)	18	16 (88.9)

Abbreviations: HF, health facility; PMT, performance monitoring team.

### Use of Information at the Health Facility Level

Similarly, improvements at health facilities were observed in using routine data for planning, target setting, quality improvement, and service delivery performance review (Table 5). The practice of using data for planning and target setting increased among most health facilities (65% in 2018 vs. 90% in 2020), while a slight change was observed in health facilities using data for analytical report production (33.3% in 2018 to 37.5% in 2020) and for supervision (51% in 2018 vs. 53% in 2020).

**TABLE 5.** Changes in Information Use and Dissemination Status in Health Facilities Between 2018 and 2020, Ethiopia

**Indicators**	**2018, %** **(N=24)**	**2020, %** **(N=24)**
HMIS data dissemination	28.6	31.2
Analytical report production	33.3	37.5
Use of data for supervision	51.0	53.1
HMIS data quality improvement	55.0	55.0
Performance review and evidence-based decision making	58.3	60.4
Planning and target setting	64.5	89.5

Abbreviation: HMIS, health management information system.

### Data Management and Processing

As Table 6 indicates, there were only slight improvements in data management and processing between 2018 and 2020. The most notable increase was in the use of data visualization (75% to 87.5%), while the use of data quality assurance mechanisms showed a slight reduction.

**TABLE 6.** Changes in Composite Indicators for Data Management and Processing in Health Facilities Between 2018 and 2020, Ethiopia

**Indicators**	**2018, %**	**2020, %**
Average score of presence of data quality assurance mechanisms	80.5	76.3
Average data analysis score	63.6	65.4
Average data visualization score	75.0	87.5
Average HIS supervision quality score	51.3	56.9
Health facilities received feedback on data quality or service performance based on HMIS data	100.0	92.3

Abbreviations: HF, health facility; HIS, health information system; HMIS, health management information system.

## LESSONS LEARNED

The CBMP partnership has created opportunities for actors from disciplines previously excluded from HIS development to contribute to ongoing improvement efforts at district and facility levels. This program promotes HIS strengthening through implementing regular gap assessments and need-based training paired with mentorship and supportive supervision visits, regular review meetings, and experience-sharing and best practice dissemination. This program also focuses on applying implementation research to learn from and improve program activities. As echoed in the literature, focusing on these types of interventions leads to HIS performance improvement. Studies conducted in Ethiopia stress the importance of providing continual in-service data management training, supportive supervision, and feedback to health workers at the primary health care unit.^15^^,^^16^ A study conducted in sub-Saharan Africa also revealed that capacity-building activities such as training, on-site mentoring, supportive supervision, and feedback mechanisms enhance knowledge, skills, and value for data, all of which contribute to behavioral change.^5^

The CBMP partnership has created opportunities for actors from disciplines previously excluded from HIS development to contribute to ongoing improvement efforts at district and facility levels.

### Changes in Data Quality

For consistent information use to occur, data need to be of good quality so that users are confident that the data they consume are complete, accurate, and available in a timely manner.^17^ Findings from this study indicate improvements in the generation and use of quality data over the 2-year intervention period. The percentage of health facilities that have a data accuracy ratio within the accepted tolerance range (±10%) improved across most tracer indicators.^13^ While encouraging, there is room for further improvement in data accuracy across all tracer indicators to ensure quality data are consistently available in the HIS. The inadequate data quality control practices and gaps in source document completeness that we observed have implications on health facility reporting accuracy. These findings are consistent with similar research conducted in other parts of Ethiopia, indicating poor quality assurance mechanisms in the country.^15^^,^^16^^,^^18^^,^^19^ Data quality assurance mechanisms succeed when they are accompanied by continual feedback, which increases the visibility of any issues and builds health workers’ capacity in addressing them.^20^ The results highlight the need for the CBMP to further build these necessary skills through tailored and targeted training, supervision, and mentorship activities. To address this gap, the program intends to institutionalize multiple levels of quality checks and feedback loops through the lot quality assurance sampling technique, routine data quality assessment, data quality desk review, and behavioral interventions that foster sustainable change in health facilities and districts.

The study results show improvements in primary health care facilities reporting aggregated routine service and disease data to higher-level facilities. Following the nationwide transition from a fragmented electronic health management information system (HMIS) to a unified, web-based District Health Information System 2 (DHIS2), intensive trainings were provided to health workers on HMIS indicator definition, data capturing, and reporting.^9^^,^^21^ The deployment of a standardized electronic data aggregation and reporting system and accompanying capacity-building activities have facilitated observed improvements in reporting completeness. Other studies have also shown that data reporting improves after an electronic, web-based HMIS platform is introduced in-country. For example, evidence from Uganda and Bangladesh showed improvement in the reporting of certain health indicators after DHIS2 implementation.^22^^,^^23^ The DHIS2 implementation in Uganda improved outpatient and inpatient reporting, as well as the reporting of health service coverage (e.g., penta 3, pregnant women who attended at least 4 antenatal care visits, and birth with a skilled birth attendant). As DHIS2 is further integrated throughout Ethiopia, we expect to see continued improvement in the reporting of health facilities within the CBMP areas.

### Transforming to an Information Use Culture in Health Facilities

We observed both positive and negative changes in the development of a data use culture during the 2-year study period. PMT meetings are one of the data use platforms institutionalized at district health offices and primary health care unit levels in Ethiopia. Findings from this study indicate that PMTs are generally used to promote the use of routine data to track performance, identify performance-related problems, and create action plans to address issues that arise. The importance of such review forums to strengthen HIS activities has been highlighted in other studies.^20^^,^^24^ While our findings show that PMTs have been established in health facilities, regular monthly PMT meetings declined over the 2-year study period. The need for social distancing because of the coronavirus disease (COVID-19) pandemic, poor Internet access to conduct meetings virtually, and shifting priorities to respond to COVID-19 may have contributed to this. For those facilities that were able to conduct meetings, encouraging progress was observed in the use of data for discussing and reviewing performance, problem identification, target setting, and planning.

Encouraging progress was observed during PMT meetings in the use of data for discussing and reviewing performance, problem identification, target setting, and planning.

This study also revealed improvements in data use for planning and target setting at health facilities. After 2 years of implementing activities under the CBMP, the use of routine health information for data presentation, root cause analysis, and action planning increased at the health facility level. This change was observed in the health facilities at the time of supervision and from success stories documented by some health facilities.^25^^,^^26^ This indicates that progress toward a data use culture among health care administrators and providers may be unfolding over time, which could contribute to improved health service provision if it continues. Classroom training is not enough to effect change, since it is not expected that all staff will apply what they learned during the training. The practical nature of mentorship coupled with trainings generated more attention and capacity to use data for action.

A culture of supportive supervision is also necessary to promote data quality and support information use at all levels of the health system. This study acknowledges the inadequacy of HIS supportive supervision practices and use of data for supervision at the primary health care level. Supportive supervision practices must be enforced to ensure higher levels of data engagement and data quality across the health system. Strengthening existing supportive supervision (in terms of frequency, quality, and approaches) can contribute to improving health workers’ capabilities by encouraging their adherence to data quality assurance practices and use of data to address problems and service delivery issues.^16^^,^^27^^,^^28^

### Data Management and Processing in Health Facilities

HIS data management and processes are also crucial to the production and use of quality data. The study revealed that some HIS processes, such as feedback loops and data visualization, are already in place at the health facility level. For managers to effectively apply facility data for the daily planning and management of primary health care delivery, the data must be processed into a meaningful format through analysis. However, results indicate that data analysis practices are weak at the health facility level. These findings are consistent with previous studies’ observations of the limited data analysis, interpretation, and problem-solving skills among health workers and how this, in turn, hinders information use at the point of care.^17^^,^^21^^,^^28^ This implies that capacity-building interventions are needed to improve the analytical skills of health workers and health facility managers. Integrating simple digital data analysis tools and dashboards as part of the CBMP intervention package, along with targeted skill development, would help address this gap.

The quarterly IR status assessment conducted by the university partners helped identify strengths and areas of improvement. Interventions were tailored based on the gaps in the assessment; behavioral interventions were also integrated, contributing to the improvements we observed. Moving forward, continual assessment and site-specific modifications are essential to further improve the effectiveness of the CBMP.

These study findings underscore the importance of maintaining regular coordination and review meetings with CBMP partners, providing focused support to facilities and district health offices, and establishing mechanisms for routine HIS improvement monitoring. Furthermore, the findings suggest that mentorship decentralization, particularly by creating and using a pool of local mentors, could increase the impact and sustainability of HIS strengthening interventions.

### Limitations

Our results should be interpreted with caution because of certain study limitations. These include the pre-post design, which lacks a control group, and the small sample size, which limits its statistical power and generalizability. While we acknowledge that our sample size is relatively small and only represents 1 district per region, sharing the results is crucial to imparting lessons learned from implementation. A more rigorous statistical evaluation of the CBMP, which will not be beset by this limitation, is forthcoming.

## CONCLUSION AND RECOMMENDATIONS

This study showed that a program focused on building health worker capacity via training and mentorship, which engages a diverse group of experts from academia and staff from various levels of government, can improve the quality and use of health data. The CBMP partnership brought together untapped expertise from local universities to contribute to the national HIS improvement efforts. Linkages between academic and implementing institutions played an enormous role in in-service training, evidence generation through collaborative research, and capacity building. Expanding this model to other settings requires clearly defining intervention packages, building the HIS expertise of the universities, and fostering partnerships between academia and districts to create local mentors. This experience can be adapted to other health sector programs as well.

## Notes

**Peer Reviewed**
